# Surface construction of fluorinated TiO_2_ nanotube networks to develop uvioresistant superhydrophobic aramid fabric

**DOI:** 10.1039/d0ra03120h

**Published:** 2020-06-12

**Authors:** Li Dong, Min Shi, Sijun Xu, Qilong Sun, Gangwei Pan, Lirong Yao, Chunhong Zhu

**Affiliations:** School of Textile and Clothing, Nantong University Nantong 226019 P. R. China xusijunwork@hotmail.com; Faculty of Textile Science and Technology, Shinshu University 3-15-1 Tokida Ueda Nagano 386-8567 Japan

## Abstract

Poor ultraviolet (UV) resistance and good hydrophilicity lead to light aging of aramid fabrics and cause heat damage to the human body. This scenario occurs when the absorbed water by the fabric evaporates and forms high-temperature water vapor in a high-temperature fire environment, which may scald the human body. Herein, a superhydrophobic hollow TNT network structure was built on surfaces of aramid fibers by surface coating fluorinated TiO_2_ nanotubes (TNTs) to develop an air-permeable, UV-protective, and superhydrophobic coating. The as-prepared superhydrophobic aramid fabric exhibited highly superhydrophobic properties against various solutions of sauce, coffee, methylene blue, active red, Au nanoparticles, Ag nanoparticles, HCl, and NaOH with liquid contact angles up to 152–160°. In addition, the superhydrophobic fabric exhibited excellent UV aging resistance (UV protection factor was 100+; 74.58% of strength retention for 24 h of UV radiation compared with 55.15% of untreated fabric), a self-cleaning function against solid soil, and original wearing characteristics, including good breaking strength and air permeability. The developed superhydrophobic coating technology may promote practical application in high-temperature environments for aramid fabrics due to its good UV resistance, chemical resistance, poromericity, superhydrophobicity, anti-fouling, and self-cleaning properties.

## Abstract 2

A superhydrophobic hollow TNT network structure was built on surfaces of aramid fibers by surface coating fluorinated TiO_2_ nanotubes (TNTs) to develop an air-permeable, UV-protective, and superhydrophobic coating.

## Graphical abstract

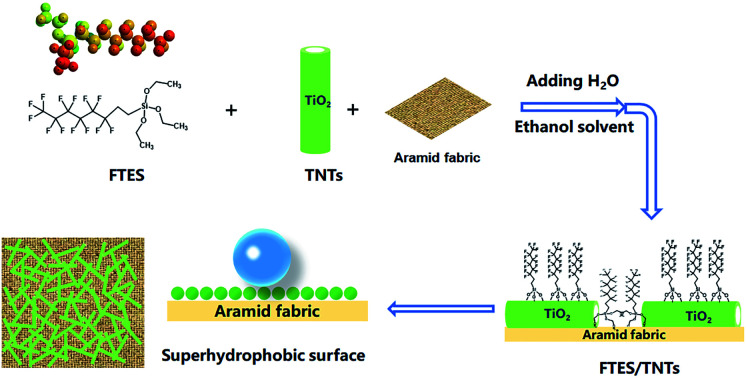

## 1. Introduction

Nature has amazing imagination and creativity, developing various delicate and complex functional structures, such as structural color of plants and insects, anti-biofouling surfaces of marine organism, echolocation system of pteropods and so on.^1–3^ Superhydrophobicity is a delicate structure developed by organisms to adapt to nature; it is manifested by various biological surfaces, such as lotus leaves, moth compound eyes, aquatic insect legs, and butterfly wings, and achieved by employing low surface energy materials and micro/nanoscale rough surfaces.^4^ The affinity of a solid material to water is quantified by the contact angle *θ* of a water droplet lying on a stationary surface. Materials with a contact angle *θ* less than 90° are defined as hydrophilic materials; otherwise, they are hydrophobic. Materials with *θ* greater than 150° are superhydrophobic.^5^ Biomimetic superhydrophobic structures have extensive application potential in self-cleaning, anti-biofouling, fluid drag reduction, anti-corrosion, and oil–water separation properties.^6–8^ With the increasing demand for high-performance functional textiles, advanced superhydrophobic textiles have received widespread attention from the upscale market due to their unique poromericity and self-cleaning ability, which are a result of their superhydrophobicity.^9,10^ Current textile researchers not only focus on traditional consumer textiles but also extend their work to special protective textiles such as moisture permeable chemical-proof clothing.^11,12^

Aramid fabrics are important wearable fire/thermal-protective materials due to their good flexibility, high temperature resistance, excellent chemical resistance, and inherent flame resistance.^13,14^ However, their light aging and hydrophilicity can cause functional decline and heat damage to wearers once liquid water absorbed by the fabric evaporates and forms high-temperature water vapor, which may diffuse into the garment and scald the skin.^15,16^ Researchers have mainly focused on the improvement of the uvioresistant performance of aramid fabric. For example, Patterson *et al.* reported a simple zinc oxide (ZnO) nanoparticle coating, which can improve the interfacial reinforcement and UV absorption of fiber-reinforced composites.^17^ Sun *et al.* developed UV uvioresistant aramid fibers by synthesizing titanium dioxide (TiO_2_) nanoparticles both on the aramid III fiber surface and in the interfacial space between the fibrils and microfibrils in supercritical carbon dioxide medium.^18^ For waterproofness, aramid fabrics are coated with an airtight waterproof coating but suffer from a significant increase in gram weight, reduction in flexibility, and poor thermal-wet comfort. Combined functions of ultraviolet (UV) resistance, waterproofness, and good air permeability are rarely reported for aramid fabric, although such features have been widely reported in natural fabrics such as cotton.^19^ By contrast, superhydrophobic coating technology offers fabrics with advanced multiple functions including poromericity, self-cleaning ability, and chemical resistance while maintaining their intrinsic advantages.^20^ The overall performance of a superhydrophobic coating is governed by its physicochemical structure including surface texture, binding forces, and surface chemistry.^5^ Given that an aramid fabric is usually exposed to high temperature, photoradiation, strong acid or base corrosion, and high oxidation conditions, its superhydrophobic coating should possess good UV, high-temperature, and chemical resistance.^4,15^ One of the most suitable methods is coating aramid fabrics with the superhydrophobic composite coating of TiO_2_ and organic hydrophobic materials. However, this method usually involves TiO_2_ nanoparticles to build a lotus leaf bionic concave–convex structure. Herein, we explore the possibility of the construction of a superhydrophobic bionic network structure by using TiO_2_ nanotubes (TNTs).

In summary, to endow aramid fabric with good properties of UV resistance, water repellency, and air permeability under an actual high-temperature scenario, superhydrophobic polyporous and hollow TNT networks on the surfaces of aramid fabric were built by impregnation with ethanol solution of TNTs and fluorinated siloxane, followed by water-triggered grafting and cross-linking reaction. The fluorinated TNT coating acted as reinforcement and wear-resistant phase displaying a uniform and porous network structure on the fiber surfaces. The superhydrophobic aramid fabric exhibited highly superhydrophobic properties against solutions of sauce, coffee, methylene blue, active red, Au nanoparticles, Ag nanoparticles, HCl, and NaOH with liquid contact angles up to 152–160° with no obvious negative influence on breaking strength and air permeability. In addition, the superhydrophobic fabric possessed improved UV resistance, UV aging resistance, and good self-cleaning function against solid soil.

## 2. Materials and methods

### 2.1. Materials

Titanium dioxide (TiO_2_) powder (average particle size of 25 nm) was purchased from Shanghai Win Chuang Degussa Co. Ltd. (China). 1*H*,1*H*,2*H*,2*H*-Perfluorooctyltriethoxysilane (FTES) was obtained from Source Leaf Biotechnology Co. Ltd. (China). Aramid fabrics were bought from Yantai Tayho Advanced Materials Co., Ltd. (China). NaOH, HCl, and ethanol were purchased from Sinopharm Chemical Reagent Co. Ltd. (China). Before the coating treatment, 16 cm^2^ square pieces of aramid fabrics were washed by toluene, deionized (DI) water, and ethanol successively to remove possible liquid and solid impurities.

### 2.2. Preparation of TiO_2_ nanotubes

Approximately 0.6 g of TiO_2_ nanoparticles was added into 60 mL of 10 M NaOH aqueous solution, followed by thermal treatment at 150 °C in a 100 mL Teflon-lined autoclave for 24 h. The autoclave was then allowed to cool to room temperature. The reaction products were centrifuged and washed with 0.1 M HCl and DI water until the pH of the rinse solution was 7.0. The final purified TiO_2_ nanotubes were dried at 60 °C and stored in a brown bottle.

### 2.3. Preparation of superhydrophobic aramid fabrics

Approximately 0.6 g of TNTs and 0.3 g of FTES were mixed in ethanol (10 mL) and ultrasonicated for 30 min. Superhydrophobic aramid fabrics were obtained by immersing 1 g of plasma-treated aramid fabric in this coating solution for 1 h, followed by adding 5 g of DI water under vigorous magnetic stirring for 2 h. The solution was dried at 60 °C for 10 min and then baked at 120 °C for 2 min.

### 2.4. Characterizations

The surface structure, morphology, and chemical composition of the fabric were characterized by using a scanning electron microscope (SEM; Hitachi 3400N, Japan) at an acceleration voltage of 8 kV and a field-emission SEM (Zeiss Gemini FESEM 300, Germany) at an acceleration voltage of 15 kV, which was equipped with an energy-dispersive X-ray spectroscope (EDS; Oxford X-Max 80). The morphological structure of TNTs was examined by using a transmission electron microscope (TEM; JEOL 2100F, Japan) at a beam acceleration of 200 kV. X-ray photoelectron spectra (XPS) were obtained by X-ray photoelectron spectroscopy (ThermoFisher Scientific, ESCALAB 250 XI, USA). The crystal structure of samples was identified by using an X-ray diffractometer (XRD; X'Pert-Pro MRD, Philips) with Cu Kα radiation (*λ* = 0.1542 nm) under a voltage of 40 kV and a current of 40 mA. The molecular chemical structure of samples was acquired using a Fourier transform infrared spectroscope (FTIR; Nicolet Instrument Corp., IS10, USA). The contact angles of aramid fabrics were measured based on the static sessile drop method by a contact angle meter (Dataphysics Instrument, OCA15EC, Germany). The ultraviolet protection factor (UPF) values of aramid fabrics were measured by a UPF detector (DaRong, 912E, China) according to the GB/T 18830-2009 standard (China). All the contact angles and UPF values were determined by averaging values measured at five different points on each sample surface. The mechanical property of fabrics was studied by a universal testing machine (Instron, Instron 5696, USA). The thermal property of samples was measured by a differential scanning calorimeter (DSC; NETZSCH, DSC-214) in a dry nitrogen atmosphere. Air permeability was investigated by using a digital air permeability measuring instrument (Darong, YG(B)461E, China).

#### Acid and base stability tests

The coated fabric was immersed in strong acid (HNO_3_, pH 2) or KOH solution (pH 14) at room temperature for 24 h. The immersed fabric was then rinsed with water and dried at room temperature for 30 min.

#### Photoaging acceleration test

The aramid fabric was placed in a photoaging test chamber (homemade) equipped with an UV light source (power: 300 W; wavelength: 365–400 nm; distance: 0.5 m) and UV radiation for 24 h.

## 3. Results and discussion

**Fig. 1:**
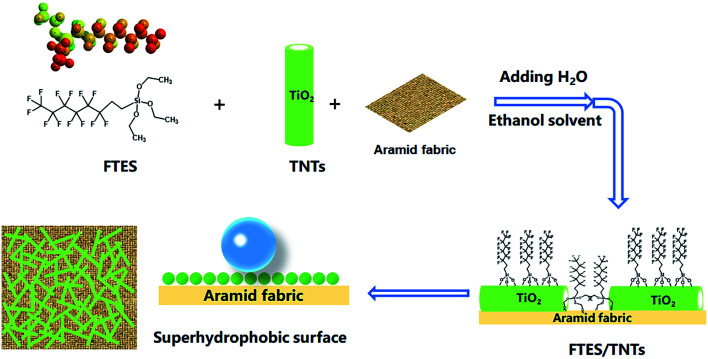
Schematic of the preparation process of superhydrophobic aramid fabrics.

Poor UV resistance and good hydrophilicity are two major weaknesses of aramid fabrics for application in a high-temperature fire environment. To address this problem, a superhydrophobic hollow TNT network structure was built on surfaces of aramid fibers by surface coating of fluorinated TNTs and FTES to develop an air-permeable, UV-protective, and superhydrophobic coating. The preparation process is illustrated in Fig. 1. Superhydrophobic aramid fabric was prepared by simple impregnation with ethanol solution of TNTs and FTES, following by adding water to trigger the grafting and polymerization reaction.

**Fig. 2:**
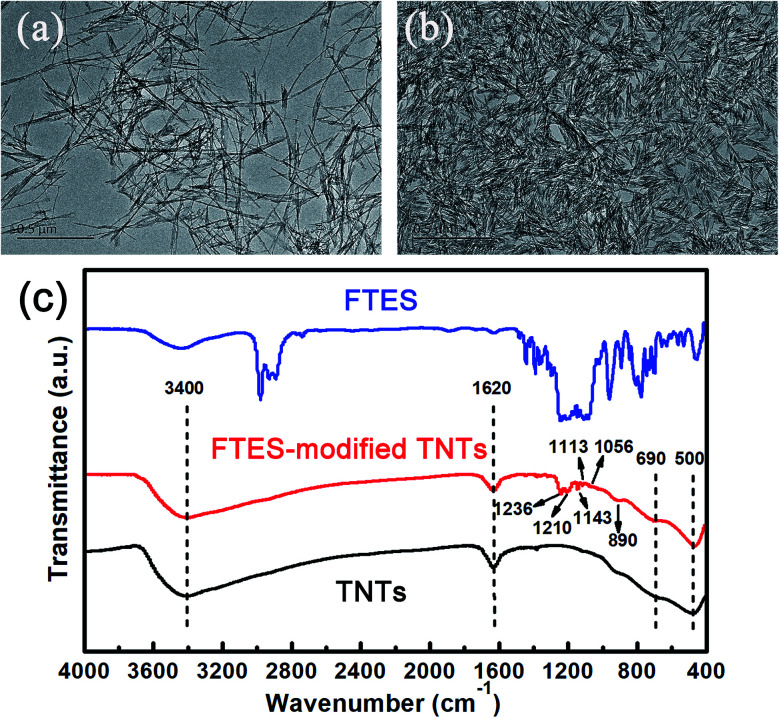
TEM images of (a) TNTs and (b) FTES-modified TNTs and (c) FTIR spectra of TNTs, FTES–TNTs, and FTES.

TNTs were synthesized according to the revised method reported in the literature.^21^ The as-prepared TNTs before modification were found to have an average tube diameter of about 8–10 nm and tube length of about 500–1000 nm, suggesting a high length–diameter ratio (as shown in Fig. 2a). Note that TNTs after modification demonstrated obvious aggregation, which could be attributed to hydrophobicity of FTES-modified TNTs (FTES/TNTs; Fig. 2b). The grafting of FTES to TNTs was evidenced by FTIR (Fig. 1c). The two convoluted peaks at around 500 and 690 cm^−1^ represented two different vibration features of the Ti–O and Ti–O–Ti bonds in the TiO_2_ lattice.^22^ A broad absorption band at around 3400 cm^−1^ and a sharp band at around 1620 cm^−1^ in all FTIR spectra for TNTs were assigned to the physically adsorbed water molecules and hydroxyl groups on surfaces of TNTs.^23^ In the FTIR spectra of FTES, the peaks at around 890, 1113, 1143, and 1236 cm^−1^ were attributed to *ν*C–F_2_ and *ν*C–F_3_ bands, respectively.^24^ By grafting FTES to TNT surfaces, FTES/TNTs inherited the typical characteristic absorption peaks of FTES. The emerging weak bands at around 1113, 1143, 1191, and 1236 cm^−1^ could be assigned to the C–F adsorption bands of FTES. In addition, two peaks at 1056 and 1210 cm^−1^ attributed to Si–O–Si indicated that FTES was grafted to TNT surfaces.^25,26^

**Fig. 3:**
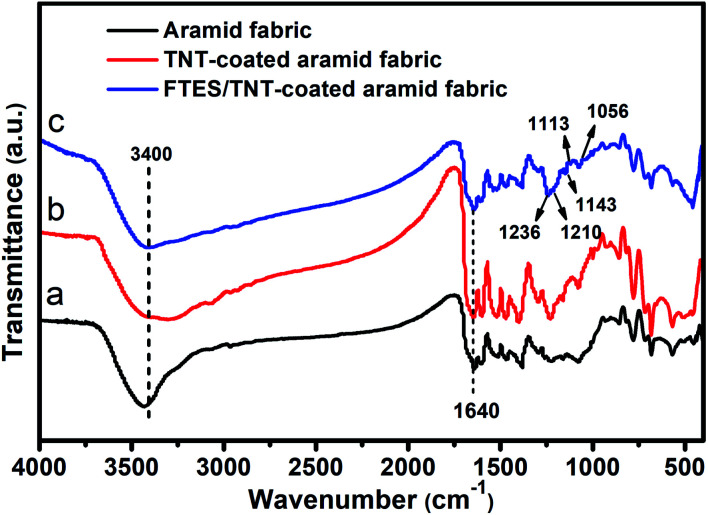
FTIR spectra of (a) pristine, (b) TNT-coated, and (c) FTES/TNT-coated aramid fabrics.

Superhydrophobic aramid fabric was prepared as described in Fig. 1. The coating of FTES/TNTs on aramid fabrics was evidenced by the FTIR spectra (Fig. 3). Aramid fabric exhibited typical strong absorption peaks of C–O stretching vibration at around 1640 cm^−1^, O=C–N and N–H deformation couple vibration at 1540 and 1260 cm^−1^, and plane vibration of N–H at 720 cm^−1^.^27^ For the FTES/TNT-coated fabric, the emerged weak absorption peaks of FTES/TNTs of the *ν*C–F_2_ and *ν*C–F_3_ bands at 1113, 1143, and 1236 cm^−1^ and Si–O–Si peak at 1056 and 1210 cm^−1^ proved that FTES and TNTs were coated to the surface of aramid fabric.

**Fig. 4:**
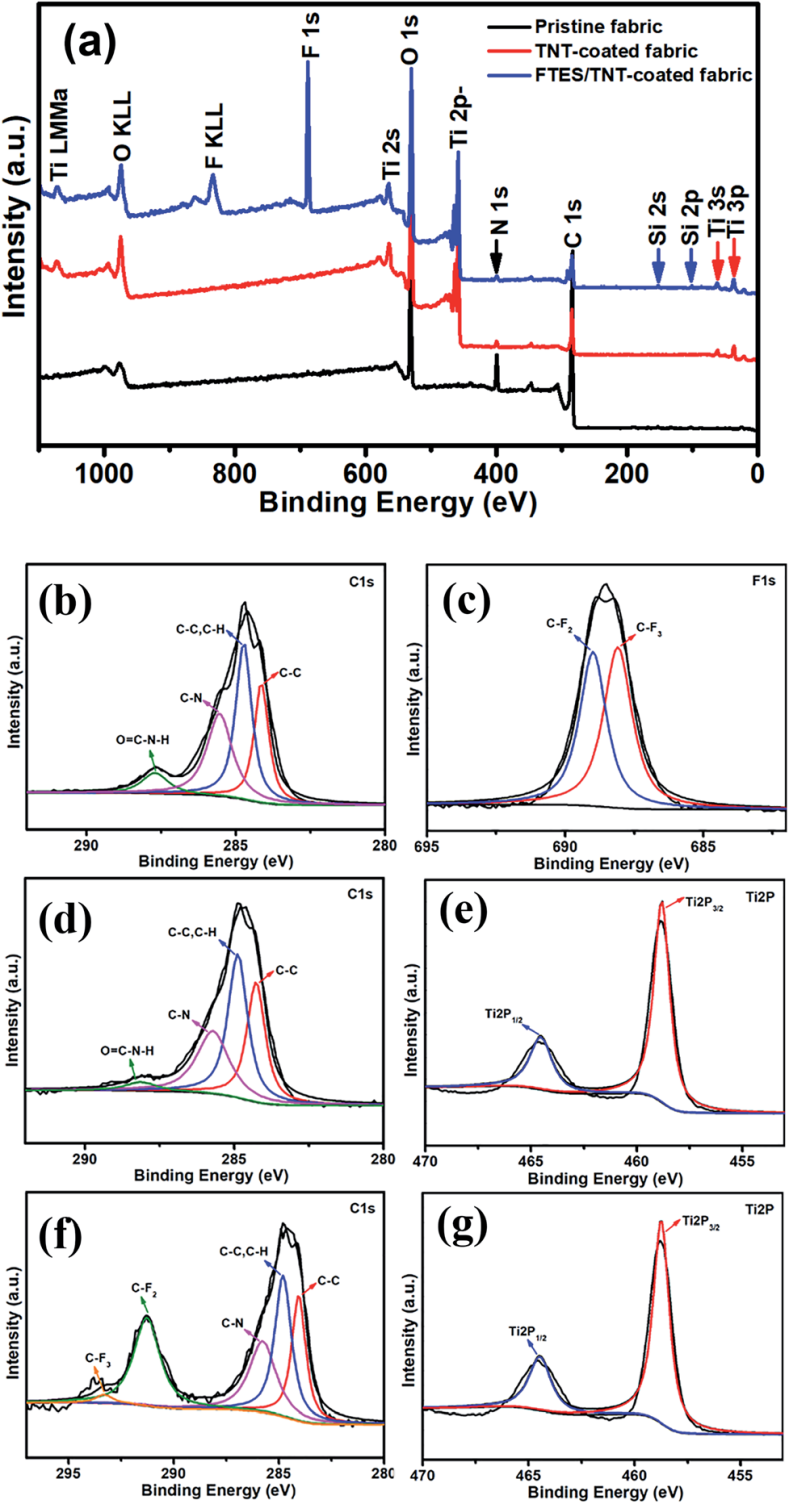
XPS spectra: (a) wide scan; C 1s spectra of (b) pristine, (c) TNT-coated, and (d) FTES/TNT-coated aramid fabric; F 1s spectra of (e) FTES/TNT-coated aramid fabric; and Ti 2p spectra of (f) TNT-coated and (g) FTES/TNT-coated aramid fabric.

**Fig. 5:**
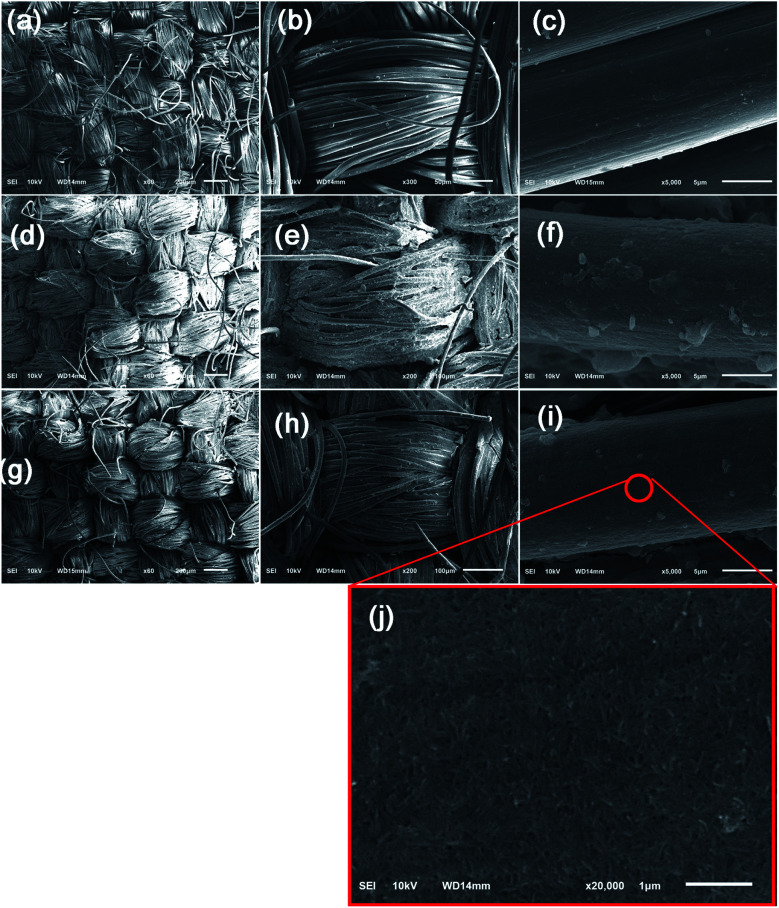
SEM images of (a–c) pristine, (d–f) TNT-coated, and (g–j) FTES/TNT-coated aramid fabrics.

The XPS survey spectra indicated that aramid fabric was composed of the elements carbon, nitrogen, and oxygen (Fig. 4a). For the TNT-coated aramid fabric, Ti LMMa, Ti 2s, Ti 3s, and Ti 3p appeared in the spectra, confirming the attachment of TNTs on the aramid fabric. By contrast, three additional strong XPS peaks of F KLL, F 1s, Si 2s, and Si 2p were detected on the FTES/TNT-coated aramid fabric, and these peaks could be assigned to the C–F and Si–O signals of FTES. Thus, aramid fabric was fully covered by FTES/TNTs. Fig. 4b–d show the fitting curves of the C 1s XPS spectra of aramid fabric, TNT-coated aramid fabric, and FTES/TNT-coated aramid fabric. The C 1s spectra of aramid fabric and TNT-coated fabric could be fitted to four independent XPS peaks of C–C, C–C/C–H, C–N, and O=C–N–H bonds with binding energies at around 284.5, 284.7, 285.5, and 287.8 eV, respectively.^28–30^ For the C 1s spectra of FTES/TNT-coated aramid fabric, besides signals of aramid fabric and TNTs, two additional ultra-strong XPS peaks of the C–F bond for FTES with a binding energy at 291.7 (–CF_2_) and 293.1 eV (–CF_3_) were separated,^31,32*o*^ thereby indicating the high content of FTES/TNTs on the surface of aramid fabric. The presence of FTES/TNTs on the surface of fabric was further confirmed by the study of the F 1s XPS signal in Fig. 5e, where the deconvolution of the fitted XPS peaks showed the molar contribution from C–F_3_ (688.2 eV) and C–F_2_ (689 eV).^33,34^ In the high-resolution Ti 2p spectra in Fig. 4f and g, the fitted XPS peaks of Ti 2p_3/2_ and Ti 2p_1/2_ were 458.5 and 464.2 eV, respectively, which were in line with the standard values of anatase TiO_2_, confirming the anatase crystalline nature of TNTs.

Fig. 5 presents FESEM micrographs of pristine, TNT-coated fabric, and FTES/TNT-coated aramid fabric. In the absence of the coating (Fig. 5a–c), the aramid fibers exhibited relatively smooth and clean surfaces with low roughness. By contrast, upon the introduction of a TNT coating, a large number of TNTs were coated to the surfaces of aramid fabric (Fig. 5d–f). However, such a TNT coating was coarse in the microscale and uneven in the nanoscale because of a lack of strong physicochemical binding force between TNTs and the fabric. For the FTES/TNT-coated aramid fabric, the surface became even and smooth in the microscale (Fig. 5g and h) due to the adhesive effect of FTES. In addition, TNTs were found to uniformly self-assemble to a dense porous network structure, thereby improving the surface roughness of the aramid fibers in the nanoscale (Fig. 5g–i). Given that the increase in surface roughness means less contact area for liquid water, this unique structure endowed the aramid fabric with excellent hydrophobicity.^35^

**Fig. 6:**
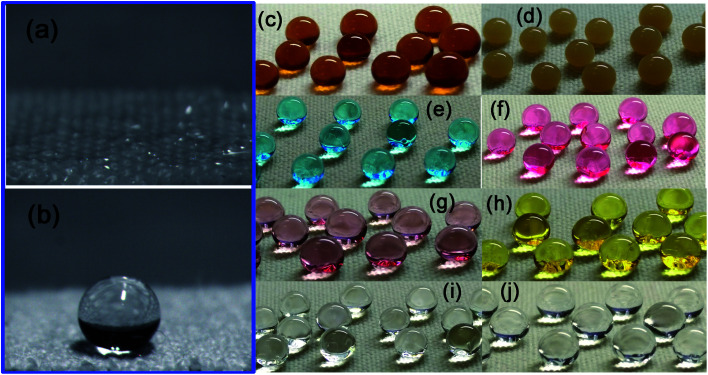
Water droplet images of the (a) pristine fabric and (b) FTES/TNT-coated aramid fabric. Photos of different aqueous droplets of (c) coffee, (d) soy sauce, (e) MB, (f) active red, (g) Au nanoparticles, (h) Ag nanoparticles, (i) HCl, and (j) NaOH on FTES/TNT-coated aramid fabric.

Water droplet images of the pristine fabric and FTES/TNT-coated aramid fabric are shown in Fig. 6a and b, respectively. When water (8 μL) was dropped onto the pristine fabric, no contact angle could be observed because the water drop completely spread and permeated into the fabric (Fig. 6a). However, when isopyknic water was placed onto the superhydrophobic fabric, a nearly sphere-like water droplet formed (Fig. 6b). Such a spherical droplet was stable and could stay supported on the fabric with the extended contact time. The water contact angle measurements revealed that the FTES/TNT-coated surface had a water contact angle of 165° and a rolling angle of 6°, indicating high superhydrophobicity. Apart from the water droplet, various aqueous solutions such as sauce, coffee, methylene blue solution, active red (AR) solution, nano Au solution, nano Ag solution, aqueous HCl (pH 1), and aqueous NaOH (pH 14) were dropped on the FTES/TNT-coated aramid fabric. As shown in Fig. 8, droplets of different solutions on the FTES/TNT-coated aramid fabric could still form a round ball, indicating the excellent super-hydrophobicity and stain resistance of the material.

**Fig. 7:**
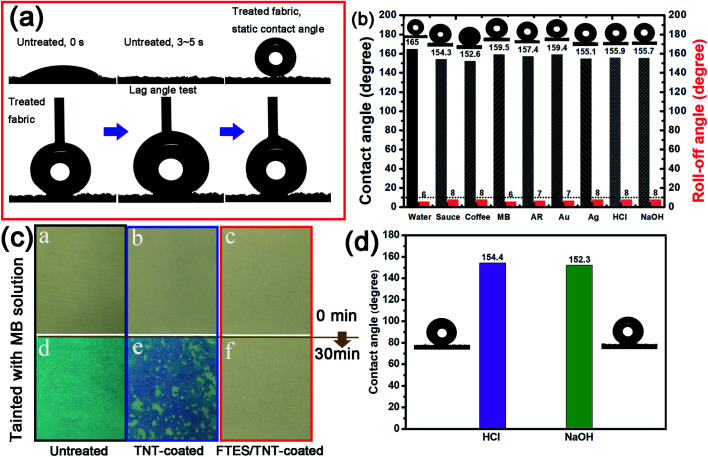
(a) Photos of the dynamic contact angles and contact lag angle test of untreated and superhydrophobic aramid fabrics. (b) The static contact and roll-off angles of solutions of sauce, coffee, MB, AR, Au NPs, Ag NPs, HCl, and NaOH. (c) Photos of color changes in untreated, TNT-coated, and FTES/TNT-coated aramid fabrics by impregnation with MB solution. (d) Contact angles of superhydrophobic fabrics with impregnation in solutions of acid and alkali for 24 h.

We also tested and calculated the lag angles of aramid fabric. The water droplet permeated into untreated aramid fabric within 5 s, indicating hydrophilicity. By contrast, the water droplet remained quasi-sphere, with a contact angle over 160° for the FTES/TNT-coated aramid fabric. The advancing and receding contact angles were 3.18° and 0.86°. Therefore, the calculated lag angle of fabric for water was 2.32°, indicating its excellent water repellency. Fig. 7b presents the contact angle values calculated using the Wenzel equation, which considers the surface roughness and penetration of liquid into grooves. Compared with sauce and coffee, MB solution, AR solution, nano-Au solution, nano-Ag solution, aqueous HCl, and aqueous NaOH had higher contact angles of 159.5°, 157.4°, 159.4°, 155.1°, 155.9°, and 155.7°, respectively. All liquid contact angles were higher than 150°, suggesting the broad-spectrum lyophobicity property. In addition, the as-prepared superhydrophobic fabrics were impregnated in an acid and alkali solution for 24 h to evaluate resistance to corrosion for the superhydrophobic coating. The contact angles of both acid and alkali solution remained largely unchanged, indicating good resistance to acid and alkaline. We also evaluated the anti-wettability of the superhydrophobic coating by impregnation of aramid fabrics in MB solution for 6 h (Fig. 7c). In line with our predictions, the untreated aramid fabric was stained with blue color with 6 h of treatment. However, the TNT-coated fabric showed a dark blue color. The hydrophilic hollow TNT network may be responsible for the enhanced adsorption of MB because of the abundant hydroxyl groups and strong nanocapillary effect. By contrast, the FTES/TNT-coated aramid fabric retained its original faint yellow hue, suggesting good antifouling property.

**Fig. 8:**
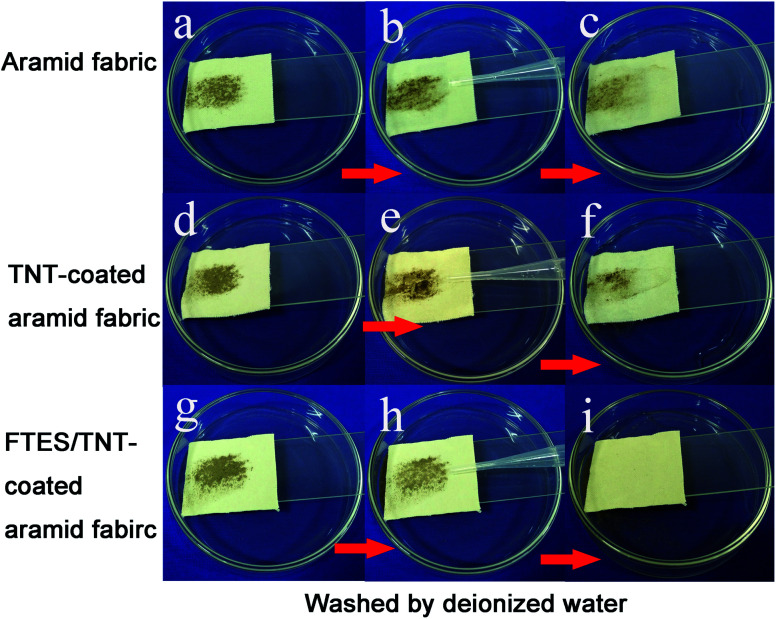
Anti-fouling and self-cleaning tests of (a–c) pristine, (d–f) TNT-coated, and (g–i) FTES/TNT-coated aramid fabrics. Samples were contaminated by solid soil, following by flushing with deionized water.

To test the self-cleaning properties of the FTES/TNT-coated aramid fabric against solid matter, soil powder was scattered over the pristine and FTES/TNT-coated aramid fabrics. When untreated aramid fabric was scoured by water drops, the soil powder mostly permeated into the fabric and only a part was removed by water because of the hydrophilicity of aramid fiber. However, water droplets could completely wash off the soil on the FTES/TNT-coated aramid fabric when the fabric was slightly inclined (Fig. 8b), suggesting the fabric's good self-cleaning performance. This characteristic could be attributed to the low surface energy on the superhydrophobic surface. Solid pollutants could be electrostatically adsorbed or dissolved by water and then roll down with water droplets.

The mechanical properties of the aramid fabrics were investigated. A comparison of the tensile strength and elongation at break among the pristine, TNT-coated, and FTES/TNT-coated aramid fabrics is summarized in Table 1. The tensile strength and elongation at break of the TNT-coated and FTES/TNT-coated aramid fabrics remained roughly unchanged, indicating that the coating of TNTs had minimal effects on the mechanical properties. In addition, the air permeability of the FTES/TNT-coated aramid fabric showed a relatively larger decline from 122.63 mm s^−1^ to 69.04 mm s^−1^, mainly because TNTs and FETS blocked a part of the inter-yarn interspace. However, the declined permeability still meets the demand of comfort requirement. Finally, no distinguishable white index change was observed for the FTES/TNT-coated aramid fabric; this effect was also beneficial for practical applications.

**Table 1:** Physical properties of uncoated, TNT-coated, and FTES/TNT-coated aramid fabric

Wearability	Pristine fabric	TNT-coated fabric	FTES/TNT-coated fabric
Tensile strength (N cm^−1^)	361 (warp)	368 (warp)	381 (warp)
	417 (weft)	450 (weft)	365 (weft)
Elongation at break (%)	25.88 (warp)	29.90 (warp)	27.64 (warp)
	17.14 (weft)	17.64 (weft)	15.39 (weft)
Air permeability (mm s^−1^)	122.63	96.76	69.04
White index (WI)	50.6	52.1	51.3

**Fig. 9:**
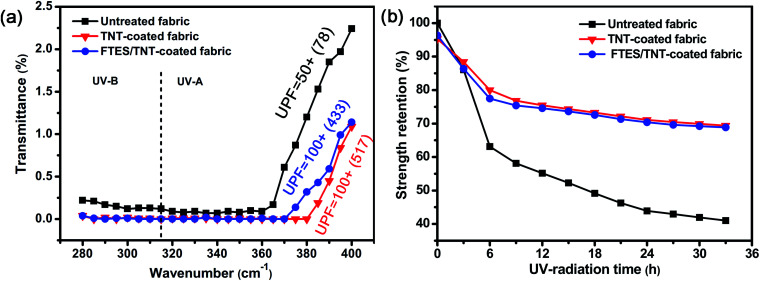
(a) UV transmittance curves of pristine and FTES/TNT-coated aramid fabrics and (b) strength retention curve of pristine and FTES/TNT-coated aramid fabrics under different UV irradiation times.

Aramid fabric is sensitive to UV light due to its highly reactive amide groups. A UV shielding coating is an effective method to retard UV photoaging. Given the strong UV absorption of TNTs, the as-prepared aramid fabric possessed good UV resistance (Fig. 9a). Compared with pure aramid fabric with UPF of around 50, the UPF of the TNT-coated superhydrophobic aramid fabrics increased up to 100+. Thus, the TNT coating blocked most of the UV radiation. In addition, the UV irradiation time-dependent strength retention tests of pristine and FTES/TNT-coated aramid fabrics indicated that the FTES/TNT-coated fabric exhibited enhanced UV light aging resistance. The strength retention of the FTES/TNT-coated aramid fabrics decreased from 77.47% (for 6 h of UV radiation) to 74.58%, 70.37%, and 68.87% (for 12, 24, and 33 h of UV radiation, respectively) compared with 63.11% (for 6 h of UV radiation) to 55.15%, 43.92%, and 40.99% (for 12, 24, and 33 h of UV radiation, respectively). The TNT-coated aramid fabric showed a similar strength retention cure to the FTES/TNT-coated fabrics, indicating that TNTs were responsible for the improved UV light resistance (Fig. 9b).

## 4. Conclusion

To endow the aramid fabric with good UV resistance, chemical resistance, poromericity, superhydrophobicity, and self-cleaning properties, FTES/TNT was prepared and coated to aramid fabric. The FTES/TNT-coated aramid fabric exhibited good superhydrophobic properties with a contact angle of 165° and a lag angle of 2.32° for deionized water. The as-prepared fabric also showed good self-cleaning properties for both solid and liquid contaminants, as well as good UV resistance (100+). The contact angles of sauce and coffee, MB solution, AR solution, nano-Au solution, nano-Ag solution, aqueous HCl, and aqueous NaOH were 159.5°, 157.4°, 159.4°, 155.1°, 155.9°, and 155.7°, respectively. These values suggested the broad-spectrum lyophobicity properties of the fabric. Acid and alkali resistance tests showed that the contact angles of the superhydrophobic fabric remained largely unchanged after impregnation in acid and alkali solution for 24 h, indicating its good resistance to acid and alkaline. Accelerated aging tests showed that the strength retention of the superhydrophobic fabric was 68.87% compared with 43.92% of untreated fabric under 33 h of UV radiation, suggesting the improvement in light aging resistance.

## Conflicts of interest

The authors declare that they have no competing interests.

## Supplementary Material
